# Paradoxical Trends in Hypertensive Heart Disease: Rising Burden in High-Sociodemographic-Index Regions Despite Healthcare Quality—An Age–Period–Cohort Analysis, 1992–2021

**DOI:** 10.3390/jcdd13080346

**Published:** 2026-07-23

**Authors:** Ngaba Neguemadji Ngardig, Jonathan N. Bella, Martial Nodjimadji Tamlengar, Amit Gulati, Anna Oneil, Riddick Osei Agyemang, Khaoula El Mardi, Gideon Gbemi, Shagun Thakur, Disha Jangra, Moiud Mohyeldin, Emamuzo Obaro Otobo, Nassim Krim, Sakshi Khurana, Imteyaz Ahmad Khan, Misbahuddin Khaja

**Affiliations:** 1 Bronxcare Health System, New York, NY 10457, USA; jbella@bronxcare.org (J.N.B.); annaoneil2022@gmail.com (A.O.); riddicko32@gmail.com (R.O.A.); khaoula.elmardi53@gmail.com (K.E.M.); adebeshop@gmail.com (G.G.); shagunthakur.ind@gmail.com (S.T.); dishaajangra@gmail.com (D.J.); mamuzeee29@gmail.com (E.O.O.); nakrim@bronxcare.org (N.K.); mkhaja@bronxcare.org (M.K.); 2 Icahn School of Medicine at Mount Sinai, New York, NY 10029, USA; 3 School of Health Sciences, Wuhan University, Wuhan 430071, China; tammartial@gmail.com; 4 Mount Sinai Morningside, New York, NY 10025, USA; dr.amitgulati@gmail.com; 5 Department of Cardiology, University of Michigan, Ann Arbor, MI 48109, USA; moiudahmed@gmail.com; 6 Massachusetts General Hospital, Boston, MA 02114, USA; 7 Columbia University Irving Medical Center, New York, NY 10032, USA; ksakshi1723@gmail.com; 8 Rutgers Robert Wood Johnson Medical School New Brunswick, New Brunswick, NJ 08901, USA; khanimteyaz001@gmail.com

**Keywords:** hypertensive heart disease, age–period–cohort analysis, mortality trends, DALYs, sociodemographic index, healthcare access

## Abstract

Hypertensive heart disease (HHD) is a major global cause of cardiovascular mortality and disability-adjusted life years (DALYs). We used an age–period–cohort model to assess mortality and DALY trends (1992–2021) across sociodemographic regions and their association with healthcare quality. Using Global Burden of Disease (GBD) data (1992–2021), we applied an age–period–cohort model to HHD mortality and DALYs in adults aged 20–54 across SDI regions and examined associations with healthcare quality. HHD mortality and DALYs declined across most SDI regions over 29 years, with high-middle SDI females showing the greatest reductions (ASMR: −3.2% annually; ASDR: −2.9%). Conversely, high-SDI regions exhibited rises of ~1.7% and ~1.2% in males and females, respectively. Low-SDI regions maintained the highest absolute burden, with 2021 mortality rates 10-fold higher in males and 13.7-fold higher in females versus high-SDI regions, despite the steepest declines. The male-to-female mortality ratio ranged from 1.74× (high-middle SDI) to 1.14× (low SDI). Age-related patterns diverged markedly: high-SDI females showed a 46.7% risk decline for ages 20–54, while males experienced a 92–111% lifespan risk increase. Period effects showed pre-2002 peak risk in lower-SDI versus post-2002 increases in high-SDI regions (males: +36%; females: +24%). The 1997–2001 birth cohort in high-SDI regions showed the highest risk (males: RR 1.75–1.85; females: RR 1.45–1.50). No significant correlation was found between HHD burden and HAQI (ASMR: r = 0.40; ASDR: r = 0.42; *p* > 0.05). HHD remains a major global health concern, with rising burden in high-SDI regions and persistent male predominance. Healthcare quality alone is insufficient, highlighting the need for targeted prevention focused on modifiable risk factors in working-age adults.

## 1. Introduction

Hypertensive heart disease (HHD) is a major global public health concern because of its substantial impact on morbidity and quality of life [1,2]. It encompasses a broad range of cardiovascular abnormalities, including left ventricular hypertrophy and both systolic and diastolic dysfunction [1,2,3,4,5]. Globally, HHD is a leading contributor to cardiovascular disease burden and health disabilities [6,7,8]. More than one billion adults are affected worldwide, and its prevalence is projected to increase from 51.2% in 2020 to 61.0% by 2050 [9].

Furthermore, chronic HHD can result in adverse cardiovascular outcomes, including heart failure, atrial fibrillation, stroke, coronary artery disease, and sudden cardiac death [2,3,10,11,12]. HHD is primarily characterized by chronic pressure and volume overload in patients with long-standing hypertension, leading to increased afterload and myocardial wall stress [13,14,15]. This process promotes pathological remodeling, most notably left ventricular hypertrophy, the hallmark feature of hypertensive cardiac damage [13,14,15].

HHD has been identified as the leading cause of cardiometabolic mortality among U.S. adults, accounting for 8.8% of all adult deaths between 2000 and 2019 [16]. China contributed more than 35% of the global increase in HHD prevalence from 1990 to 2017 [14].

A 2017 global analysis reported that Seychelles, a high-SDI country in Africa, had the highest age-standardized HHD mortality rate (59.9 per 100,000; 95% UI, 49.3–72.2) and disability-adjusted life year (DALY) rate (1082.0 per 100,000; 95% UI, 876.2–1290.9) [14]. The same study found that declines in HHD mortality and DALYs were more pronounced in high-SDI countries and slower in low-SDI countries [14]. Additionally, female sex was strongly associated with higher HHD mortality and DALYs in several countries [14]. These findings underscore the substantial and uneven global burden of HHD and highlight the need for focused public health attention.

Despite extensive research on HHD mortality, prevalence, and DALYs across various regions and countries [17,18], a key gap remains: no study has comprehensively examined HHD mortality and DALYs using an age–period–cohort (APC) model across sociodemographic index (SDI) regions from 1992 to 2021. Therefore, we conducted this study to analyze temporal trends in HHD-related mortality and DALYs across SDI-stratified regions using an APC framework. Specifically, we aimed to characterize long-term trends, quantify age-specific risks, assess period effects reflecting temporal changes in disease burden, evaluate birth cohort effects to identify generational risk patterns, and examine the association between HHD burden and the Healthcare Access and Quality Index (HAQI).

## 2. Methods

### 2.1. Data Source

HHD mortality and DALY data along with population estimates were obtained from the Global Burden of Disease (GBD) 2021 database [19], maintained by the Institute for Health Metrics and Evaluation (IHME) in Seattle, WA, USA. The GBD compiles data from multiple sources, including household surveys, censuses, vital registration systems, disease surveillance systems, and sample registration systems [19]. Rates were calculated per 100,000 in the population of males and females aged 15 years and older [20]. The GBD database also provided the HAQI values for each SDI quintile. The sociodemographic index (SDI) is a composite measure of overall sociodemographic development used within the GBD framework. It is derived as the geometric mean of three components: income per capita (lag-distributed), mean educational attainment among individuals aged ≥15 years, and total fertility rate in women aged <25 years (inversely weighted). SDI values range from 0 to 1, with countries stratified into five quintile-based tiers: low, low-middle, middle, high-middle, and high SDI. In the GBD 2021 classification, high-income countries such as the United States are categorized as high SDI, while countries such as Bulgaria and Estonia are classified as high-middle SDI.

### 2.2. Statistical Analysis

Age-standardized mortality rates (ASMRs) and age-standardized DALY rates (ASDRs) for hypertensive heart disease (HHD) were analyzed using an age–period–cohort (APC) model. In this framework, age effects reflect variations in risk across age groups, period effects capture influences affecting all age groups at a given time, and cohort effects represent differences across birth cohorts [21,22]. To disentangle these effects, age and period were decomposed into linear and nonlinear components [23], generating key parameters including net drift, local drifts, longitudinal age trends, and period and cohort deviations [24]. Net drift represents the overall annual percentage change over time. The longitudinal age curve reflects fitted age-specific rates adjusted for period effects. Period and cohort relative risks (RRs) were estimated relative to reference groups, adjusting for the remaining APC components [25].

ASMR, ASDR, and population data were grouped into consecutive five-year intervals from 1992–1996 (midpoint 1994.5) to 2017–2021 (midpoint 2019.5). Age groups ranged from 20–24 to 50–54 years. The analysis was deliberately restricted to working-age adults (20–54 years) for three reasons: (1) HHD burden in this group carries disproportionate economic and productivity consequences given its impact on the economically active population; (2) temporal trends in this age stratum are less confounded by competing causes of death that dominate in older cohorts, thereby providing a cleaner epidemiological signal; and (3) the working-age population has been systematically underrepresented in prior HHD analyses, which have predominantly focused on older adults. We acknowledge that by excluding individuals aged 55 years and older—among whom HHD burden is highest in absolute terms—our estimates represent a deliberate, focused lens on a high-priority subgroup rather than a comprehensive assessment of total population burden. Wald chi-squared tests were used to assess statistical significance (*p* < 0.01). Although APC modeling offers important advantages, it is subject to limitations, including the uncertainty principle and the identifiability problem arising from the linear dependency among age, period, and cohort (cohort = period − age) [22,25]. Analyses were conducted using the APC Web Tool (Biostatistics Branch, National Cancer Institute, Bethesda, MD, USA).

We also examined the association between HHD ASMR and ASDR net drifts and the Healthcare Access and Quality Index (HAQI) across SDI quintiles. The HAQI (range 0–100) measures healthcare system performance based on mortality from causes amenable to medical care and reflects the availability, effectiveness, and quality of health services [26].

### 2.3. Ethical Considerations

This study utilized publicly available, deidentified, population-level aggregate data from the Global Burden of Disease (GBD) 2021 database. Institutional review board approval was not required, as no individual patient data were accessed or analyzed. All primary data sources contributing to GBD underwent ethical review and approval at their originating institutions. The study adhered to GATHER and STROBE reporting guidelines.

## 3. Results

Across all SDI regions, hypertensive heart disease (HHD) mortality and disability have generally declined over the last three decades, with men consistently experiencing higher rates than women. Although the magnitude of improvement varies by development level, most regions show narrowing sex gaps and steady reductions in age-standardized mortality and DALYs. High-SDI regions are the main exception, where modest increases were observed in both sexes. Period and cohort analyses further highlight substantial long-term declines in lower-SDI regions, while associations with healthcare access and quality remain weak.

### 3.1. Trends in HHD Burden Across SDI Regions

**Figure 1:**
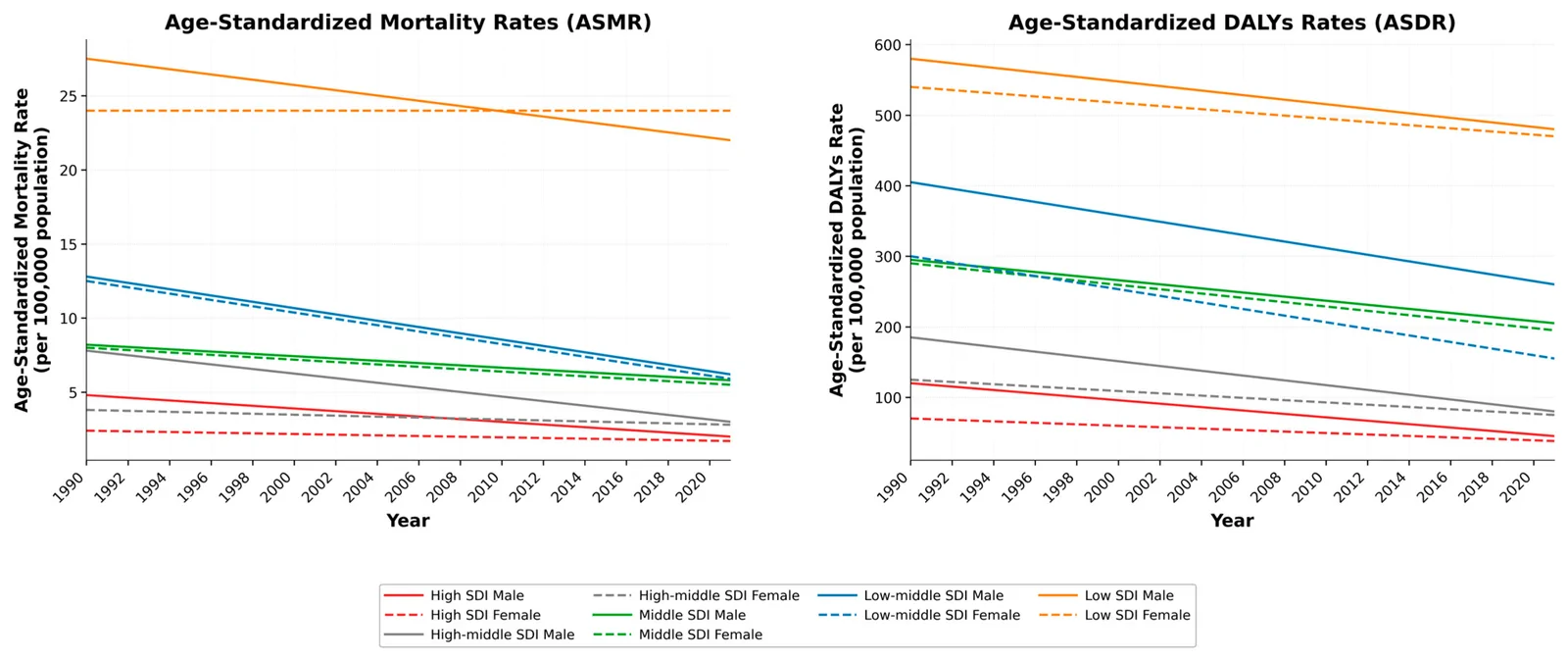
Age-standardized mortality (ASMR) and age-standardized DALY rates (ASDRs) of hypertensive heart disease (HHD) between 1992 and 2021, stratified by sex and SDI region.

Figure 1 presents 29-year trends (1992–2021) in HHD burden across five SDI regions, stratified by sex, ASMR, and ASDR. In high-SDI regions, annual declines were 0.074 per 100,000 in males and 0.04 in females and the male-to-female ratio decreased from 1.71× (4.8 vs. 2.8) in 1990 to 1.60× (2.5 vs. 1.56) in 2021. In high-middle-SDI regions, declines were 0.09 (males) and 0.039 (females), with the ratio narrowing from 1.89× to 1.74× and females showing 47% lower mortality in 2021. In middle-SDI regions, reductions were 0.10 (males) and 0.05 (females), with ratios decreasing from 1.60× to 1.50× and females having 33% lower mortality in 2021. In low-middle-SDI regions, declines were 0.10 (males) and 0.05 (females), with ratios falling from 1.42× to 1.35× and females showing 26% lower mortality. In low-SDI regions, both sexes declined by 0.10 per 100,000 per year; however, 2021 mortality remained 10× higher than high-SDI males and 13.7× higher than high-SDI females with the smallest sex gap (1.12× in 1990; 1.14× in 2021), corresponding to 12% lower mortality in females.

For HHD disability (ASDR), rates also declined in all regions, remaining higher in males. In high-SDI regions, declines were 1.84 (males) and 1.10 (females) per 100,000 per year, with ratios decreasing from 1.58× to 1.50× and females showing 33% lower disability in 2021. In high-middle-, middle-, and low-middle-SDI regions, annual declines were 2.16, 2.29, and 2.19 in males versus 1.00, 1.19, and 1.16 in females, with 2021 male-to-female ratios of 1.67×, 1.43×, and 1.29×, respectively. In low-SDI regions, declines were 2.13 (males) and 2.19 (females), yet 2021 disability remained 8.1× higher than high SDI-males and 11.2× higher than high-SDI females. The sex gap was smallest (1.07× in 1990; 1.09× in 2021), reflecting 8% lower disability in females.

Table 1 shows that among females, high-SDI regions showed slight increases in ASMR (+1.2% per year; 95% CI: −4.9 to 7.7) and ASDR (+1.1% per year; 95% CI: 0.3 to 1.9). In contrast, high-middle-SDI regions demonstrated declines in ASMR (−3.2% per year; 95% CI: −9.1 to 3.1) and ASDR (−2.9% per year; 95% CI: −3.7 to −2.2). Middle-SDI regions also showed reductions in ASMR (−2.6% per year; 95% CI: −6.6 to 1.4) and ASDR (−2.5% per year; 95% CI: −3.0 to −2.0). Similar downward trends were observed in low-middle-SDI regions (ASMR: −1.6% per year; 95% CI: −4.8 to 1.6; ASDR: −1.6% per year; 95% CI: −2.0 to −1.2) and low-SDI regions (ASMR: −1.8% per year; 95% CI: −4.1 to 0.5; ASDR: −1.8% per year; 95% CI: −2.1 to −1.5).

Among males in Table 1, high-SDI regions experienced increases in both ASMR (+1.7% per year; 95% CI: 0.3 to 1.9) and ASDR (+1.7% per year; 95% CI: 1.2 to 2.3). All other SDI categories showed declining trends: high-middle-SDI (ASMR: −2.2% per year; 95% CI: −6.8 to 2.5; ASDR: −2.1% per year; 95% CI: −2.7 to −1.5), middle-SDI (ASMR: −1.7% per year; 95% CI: −5.3 to 2.0; ASDR: −1.6% per year; 95% CI: −2.1 to −1.2), low-middle-SDI (ASMR: −1.0% per year; 95% CI: −4.4 to 2.5; ASDR: −0.9% per year; 95% CI: −1.4 to −0.5), and low-SDI regions (ASMR: −1.3% per year; 95% CI: −4.4 to 1.8; ASDR: −1.3% per year; 95% CI: −1.7 to −0.9).

### 3.2. Age Rate Ratio

**Figure 2:**
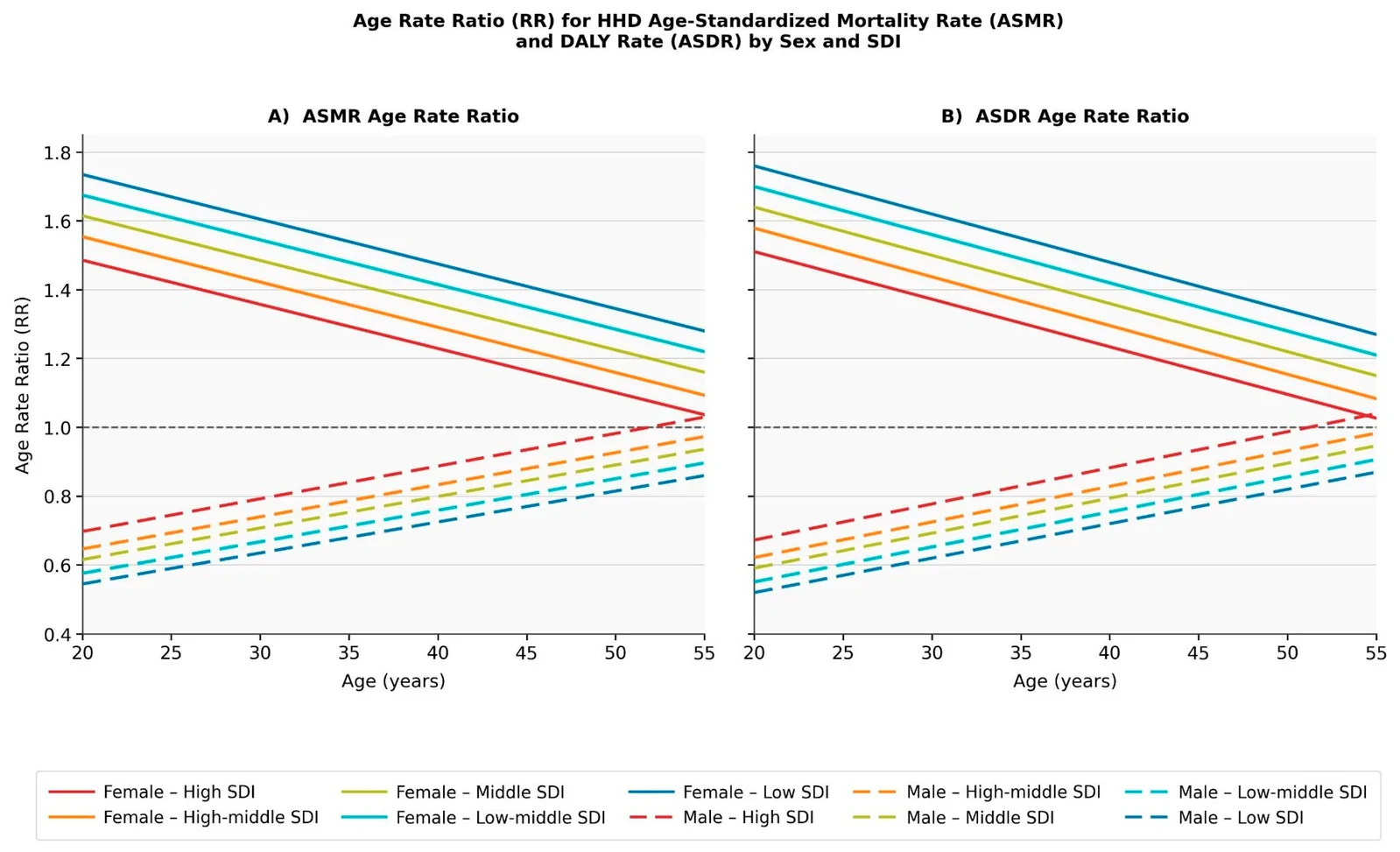
Age rate ratio (RR) of hypertensive heart disease (HHD) age-standardized mortality rates (ASMRs) and age-standardized DALY rates (ASDRs), stratified by sex and SDI region.

Figure 2 illustrates the age rate ratios (RR) for HHD ASMR and ASDR across SDI regions and sex. Age-related risk patterns diverged markedly between sexes and SDI regions. Females in high-SDI regions demonstrated a 46.7% decline in risk from age 20 to 54 years, while males in the same region experienced a 92–111% risk increase across this lifespan, with high-SDI males showing the steepest escalation.

### 3.3. Period Relative Risk

**Figure 3:**
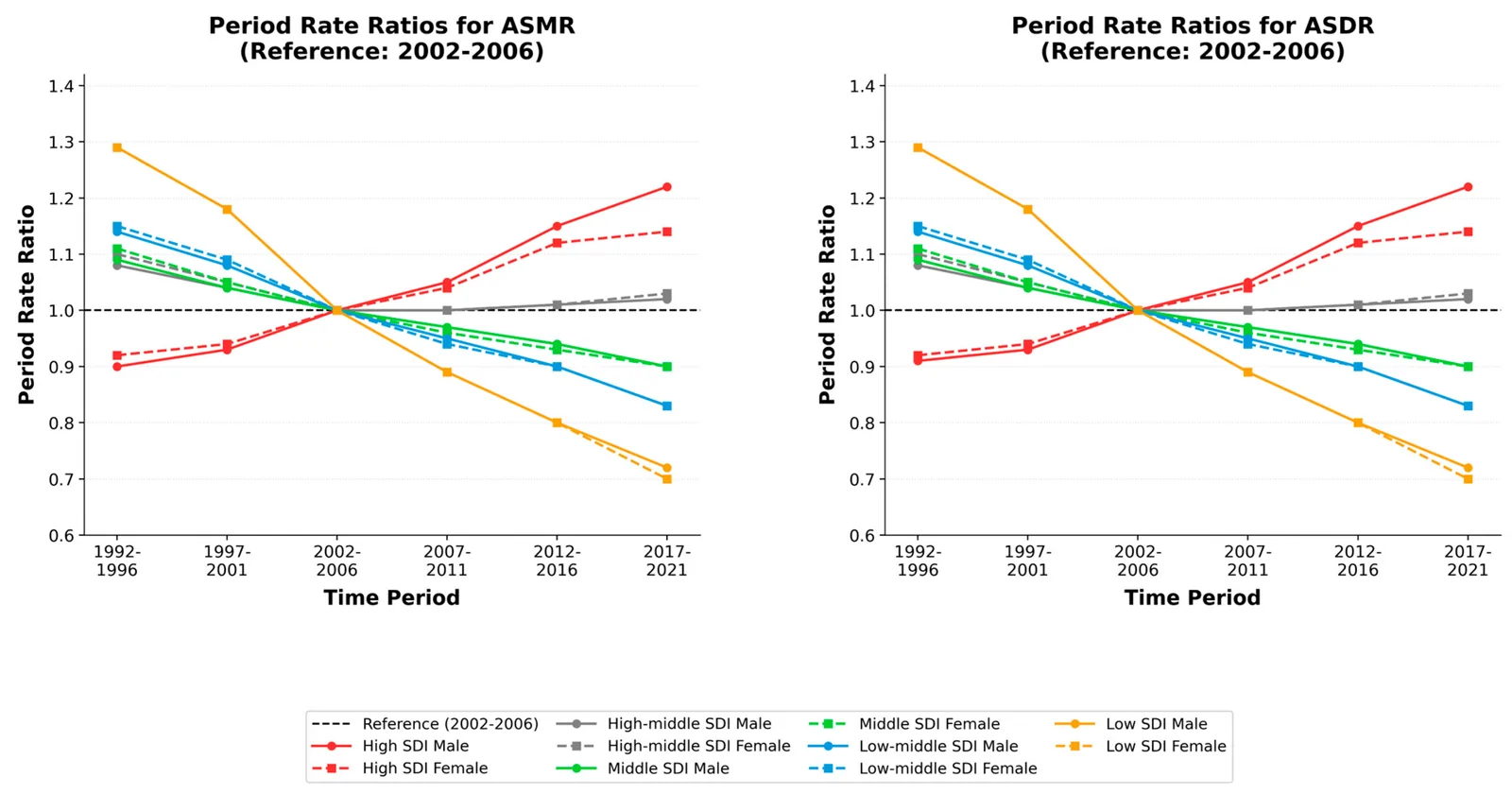
Period rate ratio (RR) of hypertensive heart disease (HHD) age-standardized mortality rates (ASMRs) and age-standardized DALY rates (ASDRs), stratified by sex and SDI region.

Figure 3 shows period rate ratios for HHD burden across six 5-year intervals (1992–2021), stratified by SDI region and sex.

In high-SDI regions, males (solid red line) experienced a 36% increase in ASMR and ASDR over 30 years, while females (dashed red line) showed a 24% increase, 12% less than males.

In high-middle-SDI regions, trends were largely stable, with a 6% increase in males (solid gray line) and approximately a 3% overall change in females (dashed gray line).

In middle-SDI regions, HHD burden declined by 20% in males (solid green line) and 17% in females (dashed green line).

In low-middle-SDI regions, reductions were more pronounced, at 29% in males (solid blue line) and 24% in females (dashed blue line).

The greatest declines occurred in low-SDI regions, with a 46% reduction in males (solid orange line) and a 44% reduction in females (dashed orange line) over the 30-year period.

### 3.4. Birth Cohort Relative Risk

**Figure 4:**
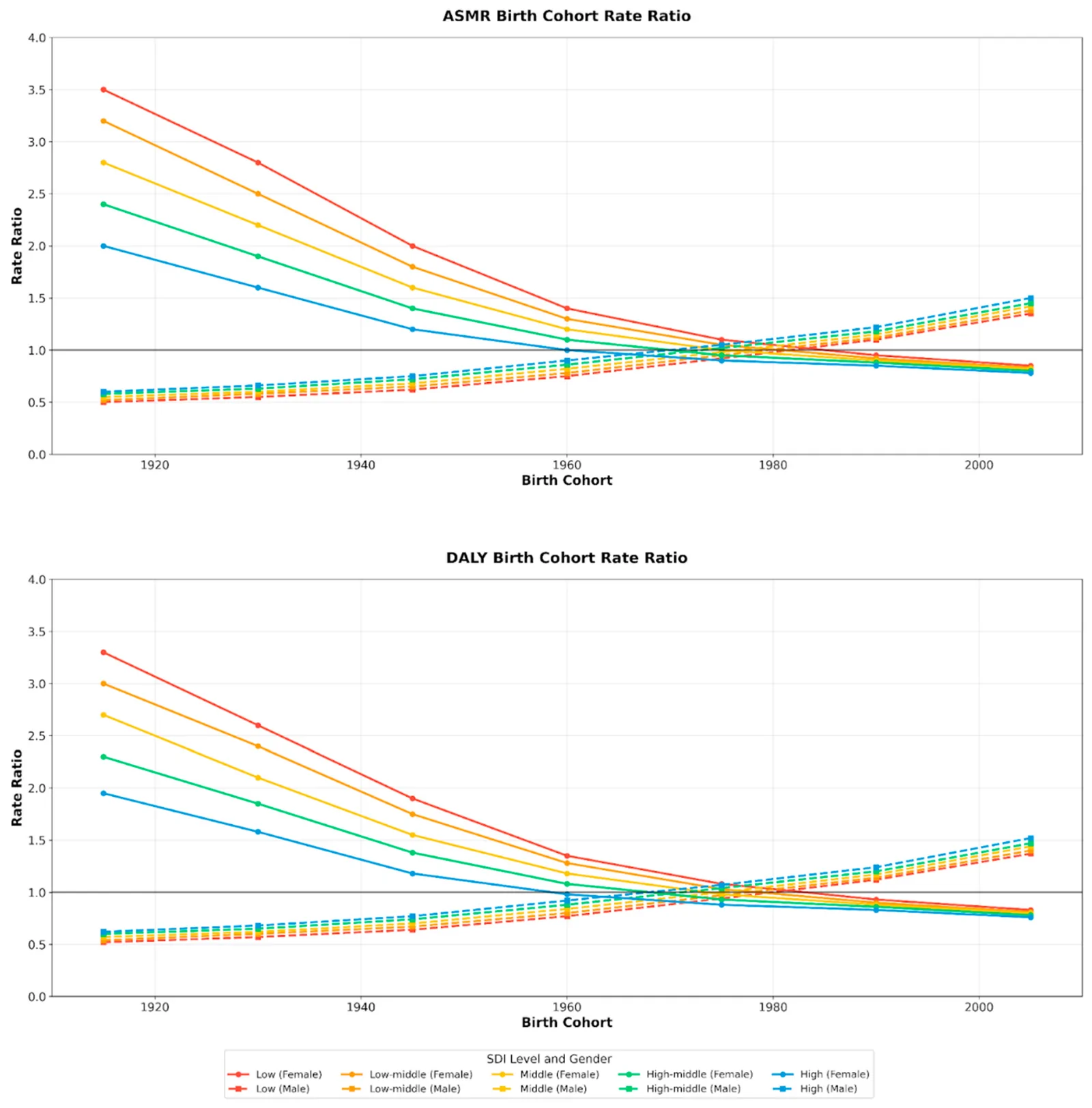
Birth cohort rate ratio of hypertensive heart disease (HHD) age-standardized mortality rates (ASMRs) and age-standardized DALY rates (ASDRs), stratified by sex and SDI region.

Figure 4 illustrates the birth cohort relative risks (RRs) for HHD age-standardized mortality rates (ASMRs) and age-standardized DALY rates (ASDRs) among women and men across different SDI regions. In the high-SDI quintile, individuals born between 1942–1946 and 1972–2001 exhibited higher risks of HHD mortality and DALYs. In contrast, in high-middle-, middle-, low-middle-, and low-SDI regions, elevated risks were observed among cohorts born between 1942 and 1971.

Within the high-SDI quintile, the lowest and highest cohort RRs were identified among those born in 1952–1956 and 1997–2001, respectively. In the remaining SDI regions, the lowest cohort RR occurred in the 1942–1946 birth cohort, whereas the highest was observed in the 1997–2001 cohort.

The most pronounced decline in cohort RRs for HHD mortality and DALYs was observed in the high-middle-SDI quintile. Specifically, HHD mortality-cohort RRs decreased by approximately 6.5-fold in females and 3.7-fold in males, while HHD DALY-cohort RRs declined by about 5.6-fold and 3.5-fold in females and males, respectively.

### 3.5. Association Between Overall Average Percentage Changes and HAQI

**Figure 5:**
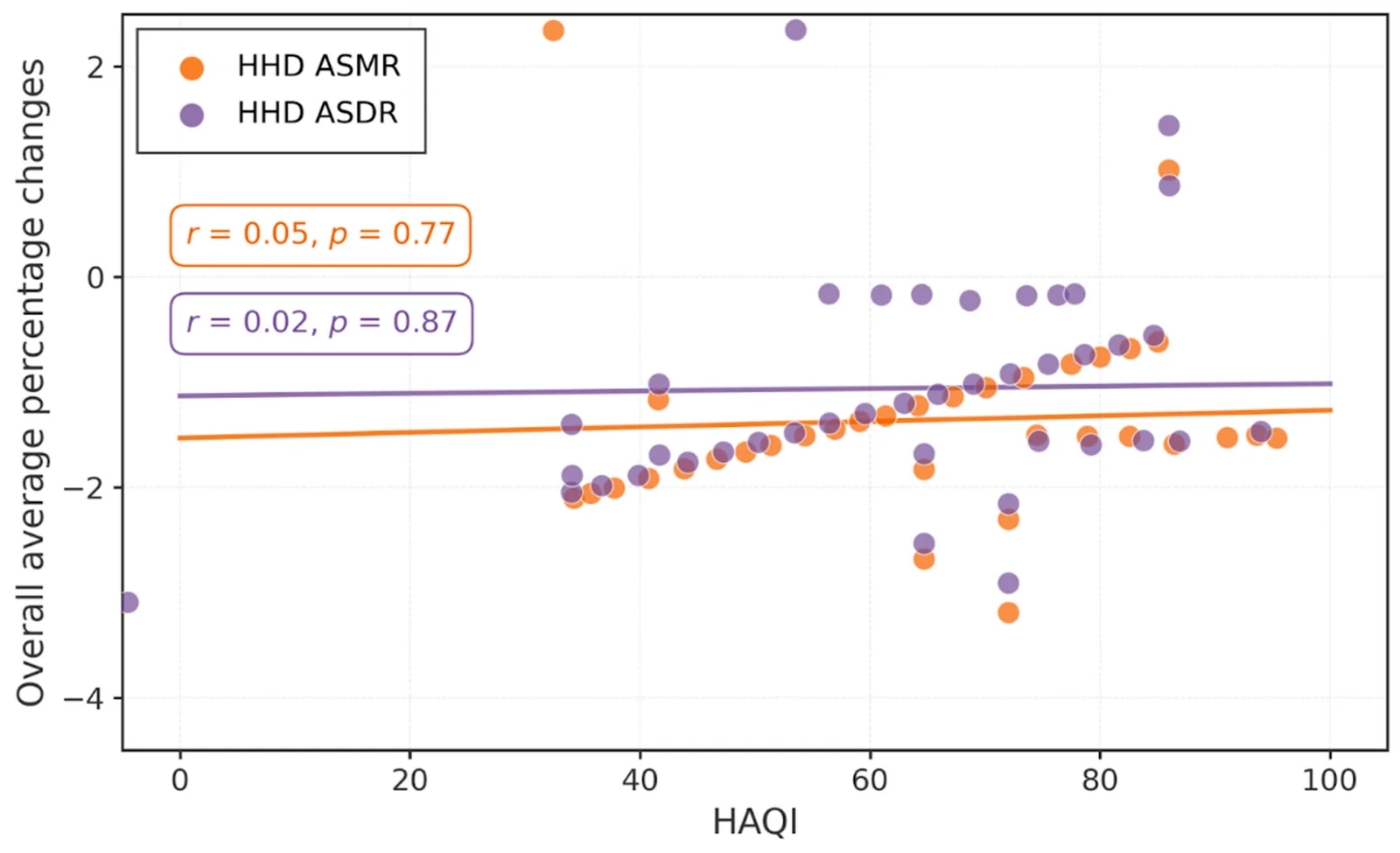
Association of the overall average percentage changes in hypertensive heart disease (HHD) age-standardized mortality rates (ASMRs) and age-standardized DALY rates (ASDRs) with the Healthcare Access and Quality Index (HAQI).

The association of HHD, ASMR, and ASDR with the Healthcare Access and Quality Index (HAQI) is represented in Figure 4. A non-significant correlation was observed between HHD mortality and HAQI. The same was noticed regarding the interaction between HHD DALYs and HAQI in the different SDI regions. Figure 5 shows that the correlation coefficient of HHD ASDR was slightly higher than ASMR (0.05, *p* = 0.77 vs. 0.02, *p* = 0.87). It is important to note that this correlation analysis was based on only five data points (one per SDI quintile), which confers critically insufficient statistical power to detect a true association. The observed non-significance therefore reflects this analytical constraint and should not be interpreted as evidence of a true null relationship between healthcare quality and HHD burden.

## 4. Discussion

This study examined trends in HHD mortality and disability-adjusted life years (DALYs), evaluating age, period, and cohort effects, as well as the association between HHD burden and the Healthcare Access and Quality Index (HAQI) across high-, high-middle-, middle-, low-middle-, and low-SDI regions. The analysis included adults aged 20–54 years from 1992 to 2021.

A key observation from this analysis is the coexistence of two distinct global patterns in hypertensive heart disease burden among working-age adults. While low- and low-middle-SDI regions continue to carry the highest absolute mortality and DALY burden, high-SDI regions demonstrate a paradoxical increase in age-standardized rates over time. This divergence suggests that absolute burden and temporal trends do not move in parallel across development strata. In lower-SDI settings, persistently high burden likely reflects structural limitations in hypertension control and healthcare access, whereas in high-SDI regions, rising trends may reflect increasing exposure to cardiometabolic risk factors despite greater healthcare availability. These findings highlight that the drivers of HHD differ across SDI levels and require context-specific prevention strategies.

Our findings indicate that female sex was consistently associated with higher HHD mortality and DALYs across SDI regions. Previous studies have similarly reported a greater burden of HHD among women compared with men [17,18]. According to the literature, women and men exhibit distinct hypertensive heart disease patterns. Women more often develop concentric remodeling and persistent hypertrophy, are more prone to diastolic dysfunction and HFpEF, and maintain higher ejection fraction (EF) during treatment; however, this cardiovascular advantage is lost when left ventricular hypertrophy (LVH) is present.

Studies [27,28,29] have found that women and men exhibit distinct patterns of hypertensive heart disease. Women more commonly develop concentric left ventricle (LV) remodeling and persistent hypertrophy and are more susceptible to diastolic dysfunction and progression to HFpEF. Targeted regression of LVH, in addition to blood pressure control, may carry greater prognostic significance in women than in men [30].

Although men tend to exhibit higher blood pressure earlier in life, the long-term cardiovascular consequences of hypertension appear more pronounced in women. Hypertension in women has been linked to physical inactivity [31] and excess caloric intake [32] and confers a higher risk of heart failure compared with men. In addition, hypertensive women develop greater arterial stiffness at older ages than hypertensive men [33,34]. Women are also more likely to have uncontrolled blood pressure with advancing age [35], and some evidence suggests that intolerance to certain antihypertensive medications may reduce treatment effectiveness in women [36].

These previous findings are consistent with our results. In our analysis, HHD mortality and DALY rates were highest in the low-SDI quintile. This pattern may reflect poorer hypertension control, fragile healthcare systems, limited diagnostic capacity, lack of affordable medications, adverse socioeconomic conditions, and high dietary salt intake, as reported in prior studies [18,37,38].

We also observed increasing trends in HHD mortality and DALYs among adults aged 20–54 years in high-SDI regions. Although the overall burden of HHD is generally lower in high-SDI settings [39], elevated burdens have been documented in certain high-middle-SDI countries, such as Bulgaria and Estonia [15]. Moreover, recent data from 2024 indicate that more than half of adults in the United States—a high-SDI country—have uncontrolled hypertension, many of whom are unaware of their condition [40]. These findings suggest that persistent and emerging risk factors in high-SDI regions may be contributing to the rising HHD mortality and DALYs trends observed in our study [14,18,41].

This paradoxical increase in HHD burden in high-SDI regions challenges the assumption that improved healthcare access alone is sufficient to reduce cardiovascular target-organ damage at the population level. One possible explanation is that indices such as the HAQI primarily reflect healthcare system performance rather than cumulative exposure to upstream behavioral and metabolic risk factors. As a result, populations in high-SDI settings may continue to experience increasing HHD burden if obesity, sedentary lifestyle, high dietary sodium intake, and suboptimal long-term blood pressure control remain prevalent. These findings suggest that treatment-centered healthcare models may be insufficient without parallel emphasis on early prevention and sustained risk-factor modification.

Overall, the decline in HHD mortality and DALY trends during the study period suggests that prevention and treatment strategies have had varying effects across SDI regions, as previously reported [20]. In addition, hereditary predisposition, tobacco use, excessive alcohol consumption, high-sodium diets, physical inactivity, urbanization, obesity, and sociocultural and economic challenges have been identified as key determinants of cardiovascular disease [42]. These factors may partly explain the heterogeneous patterns observed in hypertensive heart disease mortality and DALY trends across regions. Moreover, blood pressure control alone may be insufficient: strategies that also target regression of left ventricular hypertrophy could further reduce the overall burden.

Importantly, these findings suggest that blood pressure control alone may not fully capture the risk of hypertensive cardiac damage. HHD reflects cumulative structural and functional changes resulting from prolonged hemodynamic stress, and its burden may persist even in populations with improving hypertension awareness and treatment rates. Incorporating earlier identification of subclinical cardiac changes, particularly left ventricular hypertrophy and diastolic dysfunction, may enhance risk stratification [43] and allow for more targeted interventions in high-risk individuals.

The present study also showed that in most SDI regions, individuals younger than 30–34 years were at higher risk of HHD mortality and DALYs. In contrast, in the high-SDI quintile, the age-related risk was more pronounced among individuals older than 40–44 years. The increased burden among adults aged 20–34 years may be partly explained by the presence of risk factors that are prevalent in younger populations, such as alcohol use disorders and tobacco consumption, as reported by the World Health Organization (WHO) in 2021 [44]. There is also increasing evidence of cardiac target damage in younger individuals [45], In some cases a fifth to a third of children and young adults with primary hypertension have LVH [46].

A meaningful study has shown the validity of Cornell voltage criteria in young adults aged between 18 and 39 years [47]. Perhaps screening may be warranted to identify younger patients with cardiac target-organ damage from hypertension.

Furthermore, prior studies have consistently demonstrated that the burden of HHD increases with advancing age [14,16,39,40]. Accordingly, the higher mortality and DALY rates observed among individuals older than 40–44 years in high-SDI regions are consistent with the established age-related progression of hypertensive cardiovascular damage [18,37,38,39,40,41,42,44,48,49].

According to our findings, the period prior to 2002–2006 was associated with a higher risk of HHD mortality and DALYs among individuals aged 20–54 years in high-middle, middle-, low-middle-, and low-SDI regions. In contrast, in the high-SDI quintile, the period after 2002–2006 was linked to a higher risk of HHD mortality and DALYs in both males and females.

This aligns with evidence showing rising obesity rates during that period [50]. Additionally, increased recognition through public health initiatives [51] and improved diagnostic practices [52] may have further contributed to the observed trends. These findings represent preliminary epidemiological signals that warrant confirmation through longitudinal studies.

Regarding cohort effects, individuals born before 1967–1971 had a greater risk of HHD mortality and DALYs in high-middle-, middle-, low-middle-, and low-SDI regions. Conversely, in the high-SDI quintile, those born after 1967–1971 exhibited a higher risk. Because period effects can influence multiple age groups simultaneously, they may indirectly shape cohort patterns. Moreover, individuals from different birth cohorts experience distinct historical, environmental, and healthcare contexts, making it challenging to clearly disentangle period and cohort effects in real-world settings [26]. This interpretive challenge is compounded by the identifiability problem intrinsic to the APC framework: the perfect linear dependency defined by cohort  =  period − age means that the linear components of age, period, and cohort trends cannot be uniquely separated without an external constraint. Consequently, the net drift and local drift estimates reported here—which capture the overall and region-specific annual percentage changes in HHD burden—reflect the combined linear trajectory of all three temporal dimensions and should not be attributed exclusively to any single effect. The nonlinear deviations (period and cohort relative risks), by contrast, are identifiable and can be interpreted independently. These considerations underscore the importance of treating APC-derived linear components as descriptive epidemiological summaries that characterize the direction and magnitude of temporal change, rather than as evidence of independent age-, period-, or cohort-specific causal mechanisms.

Nevertheless, the observed variations in period and cohort relative risks likely reflect sustained national efforts to reduce the burden of HHD, as discussed in previous studies [39]. Differences in healthcare systems, therapeutic strategies, and public health policies across countries may have produced heterogeneous impacts across SDI quintiles, periods, and birth cohorts. These contextual differences may explain the distinct trends in period and cohort relative risks observed across high-, high-middle-, middle-, low-middle-, and low-SDI regions.

Our study found a non-significant positive association between HHD mortality and DALYs and the Healthcare Access and Quality Index (HAQI), with a slightly stronger correlation observed for DALYs than for mortality (r  =  0.05 vs. r  =  0.02). However, this analysis is critically underpowered: with only five observations (one per SDI quintile), the minimum detectable correlation at 80% power and α  =  0.05 exceeds r  =  0.85, rendering the analysis incapable of reliably detecting associations of small-to-moderate magnitude. The observed non-significance must therefore be interpreted with extreme caution and should not be used as a basis for policy conclusions regarding the adequacy of healthcare quality systems in reducing HHD burden. These findings highlight the need for country-level analyses with adequate sample sizes to rigorously evaluate this association. Previous studies have shown that adults experience higher cardiovascular mortality largely due to unhealthy lifestyle behaviors, including tobacco use and alcohol and opioid use disorders [42,44,48,49].

Therefore, greater emphasis should be placed on the working-age population when designing healthcare systems and public health policies. Strengthening preventive strategies and optimizing disease management in this age group could reinforce existing cardiovascular policies and contribute to meaningful reductions in HHD-related mortality and disability worldwide.

Notably, even high-SDI countries with elevated HAQI scores, such as the United States, continue to report a substantial burden of HHD among adults [40]. This observation suggests that high socioeconomic development and healthcare access alone may be insufficient to significantly curb the burden of HHD. Accordingly, further investigation into the gap between SDI and the HAQI is warranted to better understand the limited impact on HHD outcomes and to guide more targeted interventions.

### Limitations

This study has several limitations. Methodologically, a fundamental constraint of the age–period–cohort (APC) framework is the perfect linear dependency defined by the identity cohort  =  period − age, which renders the three temporal effects mathematically non-identifiable: no unique set of age, period, and cohort parameters can be estimated without imposing an external constraint or assumption (e.g., equal adjacent-cohort or adjacent-period slopes in the intrinsic estimator). This collinearity is not merely a statistical artifact, but an inherent epidemiological limitation: because age, period, and cohort effects cannot be completely disentangled—either mathematically or epidemiologically—the individual contributions of each temporal dimension to observed HHD trends remain partially confounded. Consequently, APC-derived rate ratios and net/local drifts should be interpreted as descriptive trend decompositions rather than causal estimates: they characterize the magnitude and direction of temporal change, but cannot establish whether a given trend is driven by biological aging, calendar-period exposures (e.g., shifts in treatment guidelines, screening uptake, or environmental factors), or generational cohort-specific risk profiles. Causal inference regarding the underlying determinants of the paradoxical HHD burden increase in high-SDI regions therefore requires complementary analytical approaches, such as quasi-experimental designs, interrupted time-series analyses, or individual-level longitudinal data with explicit confounder adjustment.

The analysis relied on secondary GBD 2021 data, which introduces several layers of inherent uncertainty that merit prominent acknowledgment. First, data quality is heterogeneous across SDI strata: low-SDI settings frequently depend on incomplete vital registration systems, verbal autopsies, and extrapolated estimates, which may systematically underestimate HHD burden and compress observed cross-regional differences. Second, the GBD classification of hypertensive heart disease relies on ICD-coded cause-of-death data, which are susceptible to misclassification between HHD and other hypertension-related deaths (e.g., hypertensive chronic kidney disease, ischemic heart disease with comorbid hypertension), particularly in settings where detailed death certification is limited. This misclassification may introduce non-differential measurement error that attenuates true effect sizes and reduces the reliability of cross-SDI comparisons. Third, the use of SDI-level regional aggregates, rather than country-level data, limits granularity and may mask substantial within-stratum heterogeneity, including country-specific policy environments, healthcare infrastructure, and epidemiological transitions. These data constraints should be considered when interpreting the magnitude of the observed trends and effect estimates.

The restriction to adults aged 20–54 years, while analytically deliberate, means that the oldest groups—in whom HHD incidence and absolute mortality are greatest—were excluded from the analysis. Consequently, the findings should not be generalized to the full population burden of HHD across all age strata. The study period (1992–2021) and use of SDI-level regional aggregates may mask country-specific patterns and short-term fluctuations. As a descriptive observational study, causal inferences cannot be made. Additionally, individual risk factor contributions were not quantified, confounders were not adjusted for, subgroup analyses were limited, and some estimates showed statistical uncertainty.

## 5. Conclusions

Hypertensive heart disease (HHD) remains a substantial global public health challenge, with marked disparities by sex and level of sociodemographic development. Although overall declines were observed in low- and low-middle-SDI regions, a paradoxical increase in HHD mortality and DALYs among adults aged 20–54 years was identified in high-SDI regions. Males consistently exhibited a higher burden than females, despite a narrowing sex gap over time.

Age, period, and cohort effects varied across SDI levels, reflecting shifting generational and temporal risk patterns. The lack of a significant association between HHD burden and HAQI may reflect the index’s limitation as a mortality-based surrogate of curative care that fails to capture key preventive determinants, rather than a true absence of a relationship between prevention quality and disease outcomes, underscoring the need for analyses incorporating dedicated preventive health metrics. These findings underscore the need for tailored, region-specific, and sex-sensitive prevention strategies, with greater emphasis on modifiable lifestyle risk factors and targeted interventions for working-age populations.

## Figures and Tables

**Table 1:** Net drift values (percentage) and 95% confidence intervals

Variable	HHD ASMR Net Drift (95% CI)	HHD ASDR Net Drift (95% CI)
High-SDI female	1.2 (−4.9; 7.7)	1.1 (0.3; 1.9)
High-middle-SDI female	−3.2 (−9.1; 3.1)	−2.9 (−3.7; −2.2)
Middle-SDI female	−2.6 (−6.6; 1.4)	−2.5 (−3.0; −2.0)
Low-middle-SDI female	−1.6 (−4.8; 1.6)	−1.6 (−2.0; −1.2)
Low-SDI female	−1.8 (−4.1; 0.5)	−1.8 (−2.1; −1.5)
High-SDI male	1.7 (0.3; 1.9)	1.7 (1.2; 2.3)
High-middle-SDI male	−2.2 (−6.8; 2.5)	−2.1 (−2.7; −1.5)
Middle-SDI male	−1.7 (−5.3; 2.0)	−1.6 (−2.1; −1.2)
Low-middle-SDI male	−1.0 (−4.4; 2.5)	−0.9 (−1.4; −0.5)
Low-SDI male	−1.3 (−4.4; 1.8)	−1.3 (−1.7; −0.9)

Note: Net drift represents the annual percentage change in ASMR and ASDR over the study period. Positive values indicate increasing trends, while negative values indicate decreasing trends. SDI = sociodemographic index; HHD = hypertensive heart disease; ASMR = age-standardized mortality rate; ASDR = age-standardized DALY rate.

## Data Availability

The data used in this study are publicly available from the Global Health Data Exchange (GHDx) at https://vizhub.healthdata.org/gbd-results/ (accessed on 10 June 2024).

## Abbreviations

The following abbreviations are used in this manuscript.

APC	age–period–cohort
CI	confidence interval
DALYs	disability-adjusted life years
GBD	Global Burden of Disease
GHDx	Global Health Data Exchange
HAQI	Healthcare Access and Quality Index
HHD	hypertensive heart disease
IHME	Institute for Health Metrics and Evaluation
RR	rate ratio (relative risk)
SDI	sociodemographic index
US	United States
WHO	World Health Organization
